# United States Emergency Department Screening for Drug Use Among Assault-Injured Individuals: A Systematic Review

**DOI:** 10.5811/westjem.2022.5.55475

**Published:** 2022-07-11

**Authors:** Edouard Coupet, James Dodington, Alexandria Brackett, Federico E. Vaca

**Affiliations:** 1 Yale School of Medicine, Department of Emergency Medicine, New Haven, Connecticut; 2 Yale Program in Addiction Medicine, New Haven, Connecticut; 3 Yale School of Medicine, Department of Pediatrics, New Haven, Connecticut; 4 Yale University, Harvey Cushing/John Hay Whitney Medical Library, New Haven, Connecticut; 5 University of California Irvine School of Medicine, Department of Emergency Medicine, Irvine, California

## Abstract

**Introduction:**

The clinical model of screening, providing a brief psychosocial and/or pharmacological intervention, and directly referring patients to treatment (SBIRT) is a compelling model to address drug use among assault-injured individuals in the busy emergency department (ED) setting. Our objective in this study was to examine the current literature and determine ED-based strategies that have been reported that screen, directly refer to drug mis-use/addiction specialized treatment services, or initiate addiction treatment among individuals injured by non-partner assault in the United States.

**Methods:**

We conducted a systematic review of ED-based studies using the Preferred Reporting Items for Systematic Reviews and Meta-Analyses Protocol. OVID, MEDLINE, OVID Embase, OVID AMED, Web of Science-Core Collection, Cochrane CENTRAL, and CINAHL were systematically searched using keywords and Medical Subject Heading terms. Studies were excluded if they only involved intimate partner assault-injury, tobacco, or alcohol use. We categorized ED-based strategies as screening, direct referral, or treatment initiation.

**Results:**

Of the 2,076 non-duplicated studies identified, we included 26 full-text articles in the final analysis. Fourteen studies were cross-sectional, 11 were cohort, and one was case-control in design. The most common drug use screening instrument used was the National Institute on Drug Abuse Quick Screen Question. Cannabis was the most common drug detected upon screening.

**Conclusion:**

Drug use, while highly prevalent, is a modifiable risk factor for non-partner assault-injury. The paucity of scientific studies is evidence for the need to intentionally address this area that remains a major challenge for the public’s health. Future research is needed to evaluate ED-based interventions for drug use in this population.

## INTRODUCTION

The emergency department (ED) is often referred to as the ideal setting to identify patients with high-risk health behaviors, such as substance use, and link them to evidence-based treatment services. The clinical model of screening patients, providing a brief psychosocial and/or pharmacological intervention, and directly referring them to treatment (SBIRT) has become increasingly more common in the acute care setting.^1^ The ED SBIRT, originally developed for unhealthy alcohol use, has expanded to identify and treat ED patients who report use of other substances including opioids. 2,3 Substance use is known to be associated with risk-taking related negative consequences such as injury occurrence. 4–6 As a result, more than two decades ago, the American College of Surgeons (ACS) mandated the practice of SBIRT for all trauma centers.^7^ This renders the ED an important opportunity to provide substance use treatment and potentially reduce the risk of re-injury.

Intentional injury, specifically assault-injury, presents a formidable public health burden in the United States (US). Annually, US EDs treat approximately 1.5 million individuals for non-fatal assault injuries.^8^ Previous literature reports reoccurrence rates from 1% to as high as 44%.^9^–^15^ Assault-injured individuals who report substance use are at even greater risk for re-injury. 4,15 One study demonstrated that approximately 55% of assault-injured youth compared to 40% of non-assault-injured youth in the ED have a previous history of substance use.^16^ These findings suggest that ED SBIRT may be an applicable model to identify drug use among assault-injured individuals, a population at high risk for drug use and drug use disorders, and to initiate treatment in the busy ED setting.

In this review, we sought to assess the prevalence of co-occurring drug use and non-partner assault-injury in the ED. To accomplish this, our study objective was to determine what types of ED-based strategies have been reported in the published literature that screen for drug use and/or prescription medication misuse, deliver a brief intervention that targets drug use and/or prescription medication misuse, or directly refer to specialized treatment services among individuals injured by non-partner assault, each components of the SBIRT model. We further categorized each study as to whether it evaluated screening, a brief intervention, and/or referral to specialized treatment services for drug use in accordance with the SBIRT model.

We also determined the screening method for substance use that each study used (eg, National Institute on Drug Abuse [NIDA] Quick Screen Question, “How many times in the past year have you used an illegal drug or used a prescription medication for nonmedical reasons?”). For the purposes of this study, we defined non-partner assault-injury as an intentional injury inflicted by another person not considered to be a boy/girlfriend, fiancé(e), or spouse (eg, peer, coworker, stranger). This includes individuals who may have been either the victim or aggressor. Although many studies have used the term “violence” or “violent-injury” when referring to an intentional injury inflicted by another person, in this review we will use the term “assault” or “assault-injury” for the purposes of maintaining consistency and clarity. We use the term “drug use” to refer solely to the use of drugs (eg, cannabis, cocaine) and the term “substance use” to refer to the use of both drugs and alcohol.

## METHODS

### Search Strategy

The research team developed a protocol using the Preferred Reporting Items for Systematic Reviews and Meta-Analyses Protocol (PRISMA-P) checklist.^17^ The protocol was registered in PROSPERO (registration number CRD42021270663). The searches were initially performed in June 2019 and updated in September 2021. A clinical librarian designed and executed the systematic search following a consultation with the research team using the research team’s protocol, “emergency department-based strategies that screen, refer to specialized treatment, or treat drug use and/or prescription drug misuse in assault-injured individuals: protocol for a systematic review,” as a framework. The librarian also performed a Medical Subject Heading (MeSH) analysis of pre-identified articles using the Yale MeSH Analyzer.^18^ These articles were later used to validate search concepts.

The search strategy was then peer-reviewed by another senior librarian. The search strategy used both keywords and controlled vocabulary combining the terms for drug or substance use/abuse, assault/violence or victim, and emergency department. The databases included the following: OVID Medline, OVID Embase, Web of Science, Cochrane CENTRAL, and CINAHL (See Appendix 1 search details). The final search found a total of 2177 studies with 2076 original articles. These results were exported into EndNote (Clarivate Analytics, Philadelphia, PA), where they were de-duplicated, and then uploaded to Covidence Systematic Review software (Melbourne, Australia) for screening. This study was determined to be exempt by Yale University Institutional Review Board.

### Study Selection

Two authors examined the search results for studies that screen for drug use and/or prescription medication misuse, directly refer to specialized treatment, and/or initiate ED treatment for drug use and/or prescription medication misuse among non-partner assault-injured individuals (See Table). We limited our search to literature in the US population with participants of all ages. Studies of secondary analyses were included if they reported results collected from the parent study that were deemed relevant to the study objective (eg, results of screening of drug use and/or prescription medication misuse among assault-injured individuals). Studies were excluded if they examined only intimate partner assault-injury, tobacco, or alcohol use alone. We excluded studies that examined alcohol use only to intentionally highlight knowledge gaps in the existing literature surrounding drug use and non-partner assault-injury, particularly in the setting of increasing legalization and use of cannabis.^19^

We excluded studies that examined intimate partner assault-injury only because there is a paucity of literature that evaluates drug use in non-partner assault-injury comparatively to intimate partner assault-injury. Further, we sought to intentionally identify existing knowledge gaps in the literature and inform future areas of research by consolidating the existing state of knowledge in non-partner assault-injury and drug use. All disagreements in study selection were adjudicated by a third author. After final screening of the published manuscripts, there were 26 studies used in the final analysis. The final 26 studies had substantial heterogeneity in study design, population, and main outcome. All studies were non-experimental. Of the final 26 studies, only six were prospective.

The strength of clinical data was graded according to the Oxford Centre for Evidence-Based Medicine levels of evidence, by two authors independently.^20^ Disputes were resolved after discussion. Levels of evidence are as follows: level 1, randomized clinical trials (with narrow confidence intervals) or systematic reviews (with homogeneity of randomized clinical trials); level 2, well designed controlled trials (without randomization) or prospective comparative cohort trials; level 3, case-control or retrospective cohort studies; level 4, cases series (with or without intervention) or cross-sectional studies; level 5, opinion of respected authorities or case reports.

### Data Extraction and Analysis

Data extraction was completed in full by the first author with input from the remaining authors. The identifying study information extracted included the title, first author, journal, specialty focus of journal, study funder, and year of publication. Key study information extracted included study objective, study design, study location, eligibility criteria, the instrument by which participants were screened, presence of drug use treatment, if any, referral to specialized drug use treatment, if any, and main outcomes relevant to this study’s objective. Extensive heterogeneity of the final selected studies precluded a meta-analysis. All study information was entered in tabular format in Microsoft Excel version 16 (Microsoft Corporation, Redmond, WA).

## RESULTS

### Search results

A flow chart of the study selection results can be seen in the Figure PRISMA diagram. The literature search resulted in 2,177 studies imported for screening. We identified 101 studies as duplicates and removed them, leaving 2,076 titles and abstracts. Of those abstracts, 1,984 studies (95.6%) were excluded after a title and abstract screening leaving 92 studies for full-text review. Of the 92 full-text studies, 66 studies were excluded because of wrong study design, no full-text was available (eg, conference abstracts), wrong patient population (eg, intimate partner assault-injured individuals only), wrong study setting, wrong study outcomes, or were additional duplicates. Twenty-six studies remained for the final analysis.

### Characteristics of included studies

The general characteristics and main results of the 26 studies are displayed in Appendix 2. The earliest article was published in 1999,^21^ while the most recent study was published in 2021.^22^ The journal categories of the 26 studies included the following: substance use/addiction (10/26); pediatrics (6/26); emergency medicine (5/26); public health (3/26); and medicine (2/26). Seventeen studies were funded by the NIDA,^15^,^16^,^23^–^36^ 13 by the National Institute on Alcohol Abuse and Alcoholism,^22^–^25^,^30^,^32^,^35^,^37^–^41^ eight by the US Centers for Disease Control and Prevention (CDC),^16^,^25^–^27^,^29^,^31^,^34^,^42^,^43^ one by the National Institute of Mental Health,^21^ one by the Department of Surgery at the University of Texas Southwestern Medical School,^44^ and one was not listed. 45 Fourteen studies were cross-sectional.^16^,^23^–^25^,^27^,^28^,^33^,^36^–^42^ nine were retrospective cohort,^22^,^26^,^29^–^32^,^34^,^35^,^45^ two were prospective cohort,^5^,^44^ and one was a case-control.^21^

### Study Populations

Together, the 26 study populations spanned all ages. Fourteen studies focused on both adults and adolescents, nine on adults, and three on adolescents. The mean age of the participants ranged from 14.5–38.6 years. Thirteen studies were secondary analyses of prospective studies, which were included. None of the studies were of multiple sites.

### Assessment of Substance Use

All studies screened for self-reported drug use among assault-injured participants either by computerized/written survey or in-person interview. Of the 26 studies, five studies screened for recent drug use by either survey or in-person interview without a formal screening instrument.^21^,^37^,^38^,^44^,^45^ Of the remaining 21 studies, 14 used a combination of the NIDA Quick Screen Question and Modified Alcohol, Smoking and Substance Involvement Screening Test (ASSIST),^15^,^16^,^22^,^25^–^32^,^34^–^36^ three used the Substance Abuse Outcomes Module (SAOM),^23^,^24^,^39^ two used questions from the Monitoring the Future study to detect prior-year cannabis use,^40^,^41^ one used questions from the Supporting Adolescents with Guidance and Employment survey to detect past 12-month substance use,^42^ and one used the Texas Christian University Drug Screen to determine past 30-day substance use.^33^

### Drug Use Among Assault-injured Individuals in the Emergency Department

Among all studies, drug use was found to be closely linked to assault-injury. Study results reported of this relationship were heterogenous. Four of 26 studies found a range of 25–61% of assault-injured individuals who reported drug use within the preceding 12 months.^23^,^33^,^37^,^45^ Three studies reported that previous drug use of any type was significantly associated with 1.43–7.41 greater odds of either previous or acute assault-injury.^21^,^23^,^42^ Two studies reported that assault-injury was significantly associated with 1.55–1.84 greater odds of previous drug use.^16^,^27^

### Types of Drugs Used

Overall, cannabis was the most common drug identified among assault-injured individuals. Eight studies reported cannabis use among assault-injured individuals ranged from 32.1–96.7%.^15^,^16^,^21^,^23^,^24^,^27^,^37^,^42^ Three studies found that cannabis use was significantly associated with 2.1–7.41 greater odds of assault-injury.^21^,^23^,^42^ Two studies found that cocaine use was also significantly associated with 2.7–3.1 greater odds of assault-injury.^21^,^23^ One study found prescription drug misuse was significantly associated with a 1.43 greater odds of assault-injury.^23^

## DISCUSSION

In this systematic review, we identified ED-based studies that screen, treat, and/or directly refer to specialized treatment services for drug use among assault-injured individuals. Our comprehensive literature search determined that there were 26 studies that met criteria for inclusion. The studies in this review used various screening modalities to identify drug use including an in-person interview as well as computerized and written versions of validated screening instruments for drug use. None of these studies were interventional nor did they provide a direct referral to specialized treatment services. The vast majority of studies found a high prevalence of drug use within this population, with cannabis being the most common drug detected.

Although study results were fairly heterogenous, the majority of them found high rates of drug use among assault-injured individuals, especially when compared to those injured by other mechanisms. Previous literature demonstrates a close link between assault-injury and drug use.^15^,^37^,^38^,^42^,^44^,^46^–^50^ Several pre-existing theories have explained this relationship including the shared risk factors between assault-injury and drug use, the pharmacologic effects of drug use, and the association between assault-injury and the illegal drug trade.^51^–^53^ Evidence shows that substances such as alcohol, cocaine, amphetamine-type stimulants, phencyclidine, and barbiturates cause increased aggression and impaired judgment.^51^,^54^,^55^ However, cannabis was among the most common drugs detected in our review. The evidence to support its role in causing aggressive behavior is mixed.^43^,^56^–^58^ It is more likely that the relationship between cannabis use and assault-injury is associated with the effects of withdrawal, shared risk factors of problem behavior, and facets of the illegal drug trade.^50^,^51^,^53^ Additionally, cannabis use may also allow assault-injured individuals to mitigate aggression and cope with its negative effects.^50^,^51^ Future studies are needed to better elucidate this relationship.

The practice of SBIRT to facilitate future treatment engagement for drug use in the ED setting has become increasingly common.^59^,^60^ SBIRT has shown some promise in identifying and managing unhealthy alcohol use and opioid use disorder (OUD), particularly when paired with pharmacotherapy (eg, buprenorphine for OUD).^2^,^3^ Studies in this review used various screening methods to identify drug use among assault-injured individuals. Several validated screening instruments for drug use exist, yet very few have been evaluated in the ED setting. Nineteen studies used one of the following formal screening instruments: the SAGE, SAOM, Texas Christian University Drug Screen, and the NIDA Quick Screen Question, and Modified ASSIST. The NIDA Quick Screen Question, “How many times in the past year have you used an illegal drug or used a prescription medication for nonmedical reasons?”, is likely best suited for the ED clinical care context.^1^ This single question was found to be 100% sensitive for detecting drug use in the primary care setting.^61^ Among high-risk populations such as assault-injured individuals, this instrument has the potential to be the most effective in identifying drug use in the busy ED setting.

Despite the ACS mandating the practice of SBIRT at all trauma centers for over two decades,^7^ our review demonstrates a marked paucity of literature that examines all aspects of SBIRT for drug use among assault-injured individuals in the ED setting. This includes the practices of brief intervention and/or referral to specialized treatment services for drug use. This is particularly concerning because the literature supports a strong association between non-partner assault-injury and drug use. Moreover, the COVID-19 pandemic, its associated prevention efforts, and accompanying financial stress have exacerbated both substance use and assault-injury.^62^ Yet substance use is a potentially modifiable risk factor, as evidence-based behavioral and pharmacological interventions exist.^1^,^63^

This gap in literature may be explained by the challenges of engaging the intersection of two exceptionally vulnerable populations that do not often seek healthcare with regularity.^64^,^65^ Both assault-injury and drug use are sensitive topics to research likely due to a combination of stigmatization, fear of law enforcement involvement, their shared emotional impact, and a host of other shared socioeconomic factors including poverty and racism.^48^,^66^–^69^ Furthermore, obtaining funding for assault-injury research is notoriously challenging, particularly for firearm-inflicted injuries.^70^,^71^ This may serve as an additional barrier in performing research in this vulnerable population. Other notable challenges in conducting research in this population include participant loss to follow-up by attrition (eg, unable to contact or death), undocumented immigrant status and fear of deportation, and a lack of viable and sustained community resources where patients can be referred for counseling and treatment services.^32^,^72^,^73^

Additionally, our review highlights several knowledge gaps in the existing literature surrounding drug use in the context of non-partner assault-injury. Little is known about the mutual risk factors, notably socioecological and psychological, that may contribute to the co-occurrence of assault-injury and drug use, both considered to be problem behaviors.^53^,^74^,^75^ Further, in our review, no study evaluated the potential impact of an intervention, such as a brief behavioral intervention, to reduce drug use and subsequent injury. This is particularly compelling because previous literature has shown that a brief behavioral intervention, delivered in the ED setting, demonstrates considerable promise in reducing cannabis use and its related harm as well.^76^,^77^ Future studies may use existing theory such as the social-ecological model to inform the development of an intervention that reduces the burden of drug use and injury. 78

## LIMITATIONS

The authors of the identified studies noted several limitations. One main limitation was the potential for social desirability bias in self-reported high-risk behaviors including drug use and injury mechanism due to fear of legal repercussions or embarrassment. The studies also cited small sample sizes as well as potentially limited generalizability from performing research at a single study site. Lastly, 13 of the studies included in this review were secondary analyses of two parent studies (also included in this review). Findings from these studies may also potentially limit generalizability.

## CONCLUSION

To the best of our knowledge, this review of ED-based literature that focuses on the use of screening, providing a brief intervention, and/or direct referral to specialized treatment for drug use in assault-injured individuals is the first of its kind. Existing literature included within this review supports a close relationship between non-partner assault-injury and drug use. However, results of this review highlight a substantial gap in literature that seeks to understand the complex nature of substance-use behaviors and potential interventions in this exceptionally vulnerable population. Emergency departments should consider implementing routine use of the SBIRT model to identify and treat drug use in assault-injured individuals. Areas of future investigations include ED-based interventions for drug use in this population, their potential effects on preventing re-injury, and the role that specific drugs, such as cannabis, serve in inciting aggressive behaviors and coping with its negative effects.

## Supplementary Information





## Figures and Tables

**Figure:**
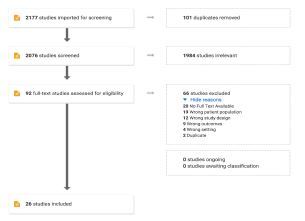
PRISMA diagram details our search and selection process applied during systematic literature search and critical review. *PRISMA*, Preferred Reporting Items for Systematic Reviews.

**Table:** Study eligibility criteria

Inclusion criteria	Exclusion criteria
US population	Intimate partner assault-injury only
All ages	Tobacco use only Alcohol use only Results of screening, direct referral to specialized treatment or initiation of emergency department treatment for drug use and/prescription medication misuse among assault-injured individuals not reported Studies outside the US

US, United States.
