# Supplementary material for: United States Emergency Department Screening for Drug Use Among Assault-Injured Individuals: A Systematic Review
Source: West J Emerg Med. 2022 Jul 11;23(4):443–50. doi: 10.5811/westjem.2022.5.55475 (PMC9391011; doi:10.5811/westjem.2022.5.55475)
Supplement: Supplementary file 2 [file wjem-23-443-s002.docx]

**Table 2. Study Characteristics and Main Results**

| Lead Author (Year Published) | Journal Category | Funder | Oxford Centre for Evidence-Based Medicine Grade | Study Objective | Study Design | Study Eligibility | Sample Size and Characteristics | ED-SBIRT Method (Instrument) | Main Results |
| --- | --- | --- | --- | --- | --- | --- | --- | --- | --- |
| Jeane Ann Grisso (1999) | Medicine | National Institute of Mental Health | 3 | To determine the correlates of violent injuries among women living in an urban area | Case-Control Study | Women aged 16-45 years presenting to one of three urban EDs in west Philadelphia for violence-related injury; control participants were women who presented for a non-violence-related injury | N=925 participants; n=405 violently-injured participants; n=520 non-violently-injured participants | Screening [Interview and Urine toxicology testing; self-reported alcohol and illicit drug use and prior treatment for alcohol and drug use; urine toxicology testing detected the presence of benzoylecgonine, cannabinoids, amphetamine and methamphetamine, opiates, and methadone] | 53% of violent injuries to women had been perpetrated by persons other than their partners; 44% of these injuries were inflicted by women, 43% by men, and 13% by both men and women; Presence of cocaine (OR 2.7 [95%CI 1.6-4.5]) and cannabis (OR 2.0 [95%CI 1.3-3.1]) were independently associated with the risk of non-partner violent injury |
| Cheryl Cherpitel (2002) | Substance Use/Addiction | National Institute on Alcohol Abuse and Alcoholism | 4 | To determine and compare the prevalence of prior 6-hour & 12-month self-reported drug & alcohol use among both injured & non-injured ED patients | Cross-Sectional Study | Patients aged ≥18 years presenting to the ED at Santa Clara Valley Medical Center in San Jose, CA | N=1,429 participants; n= 296 injured participants; n= 45 participants with violent-related injuries | Screening [Interview; self-reported drug use 6 hours & 12 months prior] | Participants with violent-related injuries were more likely to report drug use during both time periods compared to those with non-violent-related injuries; among participants with violent-related injuries, prior 6-hour drug use was as follows: 11.4% reported using a drug, 9.1% cannabis, 4.5% cocaine/amphetamine, 2.3% tranquilizers/opium/heroin/methadone, 9.1% used only one substance, 2.3% used two or more substances; prior 12-month drug use was as follows: 61% reported using a drug, 56.1% cannabis, 26.8% cocaine/amphetamine, 14.6% tranquilizers/opium/heroin/methadone, 7.7% hallucinogens/other, 36.6% used only one substance, 24.4% used two or more substances |
| Rebecca M. Cunningham (2003) | Emergency Medicine | National Institute on Alcohol Abuse and Alcoholism | 4 | To determine the amount of violence and substance abuse in an urban, academic, Level 1 trauma center in Flint, Michigan | Cross-Sectional Study | Patients aged ≥19 years presenting to ED at Hurley Medical Center in Flint, MI within 24 hours of any injury (both violent and non-violent) | N=320 participants; 51.0% female; mean age 38.6 years; 48.7% African-American, 44.5% White, 2.3% Hispanic | Screening [Interview; self-reported days of drug use in the past month] | 13.5% of participants presented with an acute violent-injury and 52.7% reported a history of violence; no significant differences were found between participants presenting with acute violence or history of violence in demographics, substance use, or substance-use related consequences; presentation of acute or history of violence was significantly associated with past month illicit drug use (OR 4.5 [95%CI 2.3-9.0]) compared to those without violence |
| Maureen Walton  (2007) | Substance Use/Addiction | National Institute on Alcohol Abuse and Alcoholism | 4 | To examine gender differences in past year violence histories and risk factors for violence among injured patients presenting to an urban ED | Cross-Sectional Study | Patients aged ≥19 years presenting to ED at Hurley Medical Center in Flint, MI within 24 hours of any injury (both violent and non-violent) | N=320 participants; 51.0% female; mean age 38.6 years; 48.7% African-American, 44.5% White, 2.3% Hispanic | Screening [Survey; self-reported drug use in the past month using the University of Arkansas Substance Abuse Outcomes Module (SAOM)] | Illicit drug use was significantly related to victimization and aggression in both men and women; men who reported generalized (non-partner & partner) aggression and victimization reported the most drug use |
| Rebecca M. Cunningham (2006) | Pediatrics | Centers for Disease Control and Prevention | 4 | To examine rates and correlates of self-reported perpetrated violence among adolescents presenting to an urban ED | Cross-Sectional Study | Patients aged 12-17 years presenting to the ED at Hurley Medical Center in Flint, MI | N=115 participants; 53.0% female; mean age 14.5 years; 51.0% African-American, 28.0% White, 9.0% American Indian or other ethnicities | Screening [Survey; self-reported substance use in past 12 months using the Supporting Adolescents with Guidance and Employment (SAGE) survey] | 77.0% of youth reported perpetrated violence in the past year; 37.0% reported severe violence (i.e. weapon use, requiring medical care), 22.0% reported cannabis use in the past 12 months; self-reported cannabis use was associated with self-reported perpetrated severe violence (OR 7.41 [95%CI 1.55-35.51]) |
| Cynthia A. Claassen (2007) | Emergency Medicine | Department of Surgery, University of Texas Southwestern Medical School | 2 | To investigate criminal and high-risk lifestyle factors that predict ED and trauma recidivism | Prospective Cohort Study | Patients aged 18-65 years, who triggered ED trauma protocol in a Level 1 urban trauma center ED in Texas | N=161 participants; 70.7% male; mean age 36.2 years; 52.4% White, 27.4% African-American, 15.2% Hispanic, 4.9% other; 21.3% victims of violence | Screening [Interview; self-reported drug use] | ED recidivism was associated with multiple substance use behaviors |
| Maureen Walton (2009) | Pediatrics | National Institute on Alcohol Abuse and Alcoholism | 4 | To examine rates and correlates of violent behaviors among adolescents presenting to an urban ED for injury or medical complaint | Cross-Sectional Study | Patients aged 14-18 years presenting to Hurley Medical Center ED in Flint, MI as a part of a larger randomized controlled trial of an intervention for alcohol use and violence | N=1,128 participants; 54.1% female; mean age 16.0 years; 58.0% African-American, 36.1% Caucasian, 5.9% other races/ethnicities; 6.0% Hispanic/Latino | Screening [Interview; self-reported cannabis use in the past year from the Monitoring the Future study] | Participants reporting severe peer violence were more likely to report cannabis use (RR 1.37 [95%CI 1.27-1.48]); no relationship between participants reporting moderate peer violence and cannabis use (RR 1.19 [95%CI 0.93-1.51]) |
| Lauren K. Whiteside (2013) | Substance Use/Addiction | National Institute on Alcohol Abuse and Alcoholism | 4 | To identify overlap and violence types between peer and dating aggression and victimization using latent class analysis among a sample of aggressive adolescents with a history of alcohol use and identify risk and protective factors associated with each violence class | Cross-Sectional Study | Patients aged 14-18 years presenting to Hurley Medical Center ED in Flint, MI as a part of a larger randomized controlled trial of an intervention for alcohol use and violence | N=694 participants; 55.6% female; mean age 16.8 years; 55.2% were African-American | Screening [Interview; self-reported cannabis use in the past year from the Monitoring the Future study] | Latent class analysis identified three violence classes: peer aggression (PA) (52.2%), peer aggression + peer victimization (PAPV) (18.6%), and multiple domains of violence (MDV) (29.3%); Compared to PAPV class, those in the MDV class had increased odds of: being female (OR 6.34 [95%CI 3.57-11.28]), being African-American (OR 3.42 [95%CI 1.92-6.08]), and using cannabis (OR 2.01 [1.10-3.68]) |
| Rebecca Cunningham (2009) | Emergency Medicine | National Institute on Alcohol Abuse and Alcoholism, National Institute on Drug Abuse | 4 | To determine the prevalence of past year non-partner violent assault and to assess variables associated with non-partner violent assault, particularly with regard to substance use | Cross-Sectional Study | Patients aged 19 to 60 years seeking care for injury or medical complaints presenting to the Hurley Medical Center ED in Flint, MI as a part of a larger randomized controlled trial linking patients to substance use treatment | N=10,744 participants; 56.0% female; mean age 36.4 years; 56.0% black, 40.0% white, 3.0% Other races; 2.0% Hispanic/Latino | Screening [Computerized Survey; Substance Abuse Outcomes Module & Diagnostic and Statistical Manual of Mental Disorders, Fourth Edition to assess for alcohol, cocaine, and cannabis abuse and dependence] | 14% reported violence in the past year; 20% of participants with past 30-day prescription drug use reported non-partner violent assault (17% reported opioid use, 7% reported prescription sedative use); 25% of those with non-partner assault noted they “got into physical fights while using alcohol or drugs” compared with 2% of those without non-partner violent assault; Reported use of: cocaine (OR 3.1 [95%CI 2.41-3.98]), cannabis (OR 2.36 [95%CI 2.02-2.76], or prescription drugs (OR 1.43 [95%CI 1.22-1.68]) increased odds of non-partner violence |
| Debra Murphy (2010) | Substance Use/Addiction | National Institute on Drug Abuse | 4 | To determine significant substance use problems concomitant in patients presenting with intentional facial injury to a Level 1 trauma center | Cross-Sectional Study | Patients aged 18 years or older who had used alcohol or drugs within the past 30 days and had at least one fracture involving the mandible, maxilla, orbit, nose, or cheekbone within the past 30 days that was due to interpersonal violence as a part of a larger randomized controlled trial | N=221 participants; 91.4% male; mean age 31.9 years; 39.4% Mexican, 26.2% Black, 10.4% White non-Latino, 9.5% Other Latino, 8.5% Multi-Ethnic, 3.2% Other, 1.8% Asian/Pacific Islander, 1.4% Native American/Alaskan | Screening [Survey and Interview; Texas Christian University Drug Screen to determine severity of drug use, substance use timeline interview to determine substance use behaviors in 30 days preceding injury] | 25% of participants screened for problem drug use |
| Stephen T. Chermack  (2014) | Substance Use/Addiction | National Institute on Alcohol Abuse and Alcoholism, National Institute on Drug Abuse | 4 | To examine clinical characteristics and treatment interests of individuals identified to have substance use disorders in an urban ED who reported past six-month history of violence or victimization | Cross-Sectional Study | Patients aged 19 to 60 years seeking care for injury or medical complaints presenting to Hurley Medical Center ED in Flint, MI who completed a 10-minute computerized survey for eligibility of a larger randomized controlled trial linking patients to substance use treatment | N=1,441 participants; 62.1% men; mean age 33.2 years; 57.6% African-American, 35.3% Caucasian, 2.4% Hispanic/Latino, and 4.7% Other | Screening [Computerized Survey; Substance Abuse Outcomes Module & Diagnostic and Statistical Manual of Mental Disorders, Fourth Edition to assess for alcohol, cocaine, and cannabis abuse and dependence] | Participants who reported both partner and non-partner violence had increased odds of a diagnosis of cocaine abuse/dependence (OR 2.35 [95%CI 1.45-3.80]); participants who reported both partner and non-partner victimization had increased odds of a diagnosis of cocaine abuse/dependence (OR 2.27 [95%CI 1.43-3.59]) |
| Rebecca M. Cunningham (2015) | Pediatrics | National Institute on Alcohol Abuse and Alcoholism, National Institute on Drug Abuse, the Centers for Disease Control and Prevention | 2 | To examine 2-year outcomes of fatal and non-fatal violent injuries among a sample of assault-injured, drug-using (AI) youth seeking ED care compared with non-assault injured, drug-using (non-AI) youth seeking ED care for other reasons | Prospective Cohort Study | Patients aged 14-24 years presenting to Hurley Medical Center ED in Flint, MI for assault-related injury and a non-assault-related injury group proportionally balanced by sex and age who self-reported any drug use | N=599 participants; 58.8% male; mean age of AI group 20.1 years, mean age non-AI group 20.0 years; 58.3% African-American | Screening [Computerized Survey; Drug Abuse Treatment Outcome Studies, NIDA Quick Screen Question & Modified Alcohol, Smoking and Substance Involvement Screening Test (ASSIST)] | AI group had twice the risk for violent injury compared to non-AI group (36.7% vs. 22.4%; RR 1.65 [95%CI 1.25-2.14]); Female sex (RR 1.30 [95%CI 1.02-1.65]), assault-related injury (RR 1.57 [95%CI 1.19-2.04), diagnosis of a drug use disorder (RR 1.29 [95%CI 1.01-1.65] at index visit were predictive of ED recidivism or death at 24 months; assault-related injury (p<.001) and diagnosis of drug use disorder (p=.03) shortened expected waiting time until the first ED re-visit for violence or death |
| Patrick Carter (2013) | Pediatrics | National Institute on Alcohol Abuse and Alcoholism, National Institute on Drug Abuse, the Centers for Disease Control and Prevention | 4 | To determine firearm possession rates and associated correlates among youth seeking care for assault in an ED and to understand differences in risk factors among these youth seeking care with and without firearm possession | Cross-Sectional Study | Patients aged 14-24 years presenting to Hurley Medical Center ED in Flint, MI for assault-related injury and a non-assault-related injury group proportionally balanced by sex and age who self-reported any drug use | N=689 participants; n=159 participants with firearm possession; n= 530 participants without firearm possession; 50.4% male; mean overall age 19.8 years; 60.8% African-American | Screening [Computerized Survey; Drug Abuse Treatment Outcome Studies, NIDA Quick Screen Question & Modified Alcohol, Smoking and Substance Involvement Screening Test (ASSIST)] | Past 6-month illicit drug use was positively associated with firearm possession (66.7% vs 49.3%, p<0.001); participants with firearms reported higher rates of prescription opioid use before a physical fight (19.5% vs 11.3%, p<0.01) |
| Rebecca Cunningham (2014) | Pediatrics | National Institute on Drug Abuse, Centers for Disease Control and Prevention | 3 | To characterize youth seeking care for assault injuries, the context of violence, and previous ED service utilization to inform ED-based injury prevention | Cross-Sectional Study | Patients aged 14-24 years presenting to Hurley Medical Center ED in Flint, MI for assault-related injury and a non-assault-related injury group proportionally balanced by sex and age who self-reported any drug use | n=718 participants in assault-related injuries (AI) group; n=730 participants in comparison (non-AI) group; 50.8% male in AI group; 51.6% male in non-AI group; mean age AI group: 19.7 years; mean age comparison group: 19.6 years; 60.7% African-American in AI group; 56.0% African-American in non-AI group | Screening [Computerized Survey; Drug Abuse Treatment Outcome Studies, NIDA Quick Screen Question & Modified Alcohol, Smoking and Substance Involvement Screening Test (ASSIST)] | AI group reported higher prior ED utilization for assault than non-AI group (OR 2.16 [95%CI 1.38-3.38]); AI group reported having greater odds of substance misuse than non-AI group (OR 1.84 [95%CI 2.26-4.48]) |
| Patrick Carter  (2015) | Pediatrics | National Institute on Drug Abuse, Centers for Disease Control and Prevention | 3 | To describe rates of firearm aggression, victimization, and fatal/nonfatal firearm injury during the 2 years after an ED visit for assault and to examine modifiable predictors of this firearm violence | Retrospective Cohort Study | Patients aged 14-24 years presenting to Hurley Medical Center ED in Flint, MI for assault-related injury and a non-assault-related injury group proportionally balanced by sex and age who self-reported any drug use | n=349 participants in assault-related injuries (AI) group; n=250 participants in the comparison (non-AI) group; n=70 participants in the AI group sustained a firearm-related injury at baseline | Screening [Computerized Survey; Drug Abuse Treatment Outcome Studies, NIDA Quick Screen Question & Modified Alcohol, Smoking and Substance Involvement Screening Test (ASSIST)] | Participants determined to have a drug use disorder 6 months prior to baseline ED visit were at greater risk for sustaining an injury from firearm-violence after 24 months (RR 1.40 [95%CI 1.16-1.69]) |
| Kipling M. Bohnert (2015) | Substance Use/Addiction | National Institute on Drug Abuse, Centers for Disease Control and Prevention | 4 | To compare assault-injured youth in an urban ED who reported drug use, with a systematically sampled comparison group of youth who presented to the ED for other medical reasons and also reported drug use, in order inform future potential interventions aimed at addressing unmet substance use and mental health service needs | Cross-Sectional Study | Patients aged 14-24 years presenting to Hurley Medical Center ED in Flint, MI for assault-related injury and a non-assault-related injury group proportionally balanced by sex and age who self-reported any drug use | n=350 participants in AI group; 250 participants in comparison (non-AI) group; 41.2% female overall; mean overall age: 20.1 years; 58.2% African-American | Screening [Computerized Survey; Drug Abuse Treatment Outcome Studies, NIDA Quick Screen Question & Modified Alcohol, Smoking and Substance Involvement Screening Test (ASSIST)] | 57% of participants overall met criteria for drug use disorder, only 9% received prior treatment; AI group had an increased odds of reporting any drug use (OR 1.55 [95%CI 1.11-2.16]), cannabis use (OR 1.46 [95%CI 1.05-2.04]), or illicit drug use (excluding cannabis) (OR 2.70 [95%CI 1.77-4.11]) within prior day of index ED visit compared to non-AI group |
| Jason E. Goldstick (2016) | Substance Use/Addiction | National Institute on Drug Abuse | 4 | To identify characteristic substance misuse profiles among youth entering an urban ED and explore how those profiles relate to individual- and community-level factors | Cross-Sectional Study | Patients aged 14-24 years presenting to Hurley Medical Center ED in Flint, MI who completed a screening survey as a part of a larger cohort study | N=878 participants; 55.5% presented to the ED for violent injury; 50.7% female; mean age 19.7; 71.6% African-American | Screening [Computerized Survey; Drug Abuse Treatment Outcome Studies, NIDA Quick Screen Question & Modified Alcohol, Smoking and Substance Involvement Screening Test (ASSIST)] | Four groups of participants by substance use were identified: non-misuse (51.5%), cannabis-only (27.9%), alcohol/cannabis (16.1%), and multiple substances (4.6%); compared to the non-misuse group, each of the three remaining groups had higher rates of serious violence |
| Patrick Carter (2017) | Public Health | National Institute on Drug Abuse, Centers for Disease Control and Prevention | 3 | To utilize timeline followback data to characterize the circumstances surrounding non-partner firearm violence | Retrospective Cohort Study | Patients aged 14-24 years presenting to Hurley Medical Center ED in Flint, MI for assault-related injury and a non-assault-related injury group proportionally balanced by sex and age who self-reported any drug use | n=143 participants reported a firearm-related non-partner conflict during the 24-month follow up period; n=278 participants reported a non-firearm-related non-partner conflict during the 24-month follow up period | Screening [Computerized Survey; Drug Abuse Treatment Outcome Studies, NIDA Quick Screen Question & Modified Alcohol, Smoking and Substance Involvement Screening Test (ASSIST)] | Participants who reported cannabis use at baseline ED visit were at increased odds of having a firearm-related conflict after 24 months (OR 1.82 [95%CI 0.37-8.88]); Reported previous illicit drug use and diagnosis of drug use disorder at baseline ED visit were not associated with firearm conflict after 24 months |
| Maureen A. Walton  (2017) | Substance Use/Addiction | National Institute on Alcohol Abuse and Alcoholism, National Institute on Drug Abuse | 3 | To examine factors associated with greater cannabis use trajectories among drug-using youth presenting to the ED | Retrospective Cohort Study | Patients aged 14-24 years presenting to Hurley Medical Center ED in Flint, MI for assault-related injury and a non-assault-related injury group proportionally balanced by sex and age who self-reported any drug use | n=599 participants; 58.8% male; mean age 20.1 years; 58.3% were African-American, 32.5% Caucasian. 9.3% other | Screening [Computerized Survey; Drug Abuse Treatment Outcome Studies, NIDA Quick Screen Question & Modified Alcohol, Smoking and Substance Involvement Screening Test (ASSIST)] | Five cannabis use trajectory groups were identified: low, intermittent, moderate decline, high decline, and chronic; At baseline ED visit, male participants and those reporting greater physical aggression were at greater odds for being in the intermittent-, moderate decline-, high decline-, and chronic-cannabis use groups compared to the low-cannabis use group |
| Patrick Carter (2018) | Public Health | National Institute on Drug Abuse, Centers for Disease Control and Prevention | 3 | To characterize arrests among high-risk, assault-injured, drug-using youth following ED treatment | Retrospective Cohort Study | Patients aged 18-24 years presenting to Hurley Medical Center ED in Flint, MI for assault-related injury and a non-assault-related injury group proportionally balanced by sex and age who self-reported any drug use who participated in a larger cohort study | n=299 participants in the assault-related injuries (AI) group; n=212 participants in the comparison (non-AI) group; 57.1% male; 58.3% black | Screening [Computerized Survey; Drug Abuse Treatment Outcome Studies, NIDA Quick Screen Question & Modified Alcohol, Smoking and Substance Involvement Screening Test (ASSIST)] | Participants in the AI group had a greater risk of arrest than the non-AI group (38.1% vs 25.9%; RR 1.47 [95%CI 1.07-2.02]); Participants who were arrested after 24 months had a greater odds of being: male (RR 1.56 [95%CI 1.35-1.79]), present with an assault injury (RR 1.24 [95%CI 1.08-1.44), use cannabis (RR 1.03 [95%CI 1.00-1.06]), and meet criteria for a drug use disorder (RR 1.32 [95%CI 1.13-1.52]) during their baseline ED visit |
| Jessica S. Roche (2018) | Emergency Medicine | National Institute on Drug Abuse, National Institute on Alcohol Abuse and Alcoholism, Centers for Disease Control and Prevention, National Center for Advancing Translational Sciences, and National Institute of Child Health and Development | 3 | To describe the methods for retention in youth violence studies and the characteristics of hard-to-reach participants | Retrospective Cohort Study | Patients aged 14-24 years presenting to Hurley Medical Center ED in Flint, MI for assault-related injury and a non-assault-related injury group proportionally balanced by sex and age who self-reported any drug use | n=349 participants in the assault-related injuries (AI) group; n=250 participants in the comparison (non-AI) group; 58.8% male; 58.2% African-American | Screening [Computerized Survey; Drug Abuse Treatment Outcome Studies, NIDA Quick Screen Question & Modified Alcohol, Smoking and Substance Involvement Screening Test (ASSIST)] | Participants presenting with: assault injury (Incident Rate Ratio (IRR) 0.906 [95%CI 0.821-1.000]) or a substance use disorder (IRR 0.861 [95%CI 0.779-0.952]) during their baseline ED visit required fewer contact attempts to complete their 24-month follow-up |
| Jason E. Goldstick (2019) | Medicine | National Institute on Alcohol Abuse and Alcoholism and National Institute on Drug Abuse | 3 | To determine the prevalence of alcohol use disorder and the frequency of alcohol use disorder transitions | Retrospective Cohort Study | Patients aged 14-24 years presenting to Hurley Medical Center ED in Flint, MI for assault-related injury and a non-assault-related injury group proportionally balanced by sex and age who self-reported any drug use | n=599 participants; 58.8% male; mean age 20.1 years; 58.3% were African-American, 32.5% Caucasian. 9.3% other | Screening [Computerized Survey; Drug Abuse Treatment Outcome Studies, NIDA Quick Screen Question & Modified Alcohol, Smoking and Substance Involvement Screening Test (ASSIST)] | Cannabis use severity both hastened alcohol use disorder onset (HR 1.18 [95%CI 1.06-1.32]) and slowed alcohol use disorder remission (HR 0.85 [95%CI 0.76-0.95]) |
| Sarah A. Stoddard (2020) | Substance Use/Addiction | National Institute on Drug Abuse and Centers of Disease Control and Prevention | 3 | To describe the non-partner violence patterns of high-risk youth presenting to an urban ED over 24 months and to examine baseline covariates of non-partner violence patterns, with particular examination of the relative contribution of substance use | Retrospective Cohort Study | Patients aged 14-24 years presenting to Hurley Medical Center ED in Flint, MI for assault-related injury and a non-assault-related injury group proportionally balanced by sex and age who self-reported any drug use | n=599 participants; 58.8% male; mean age 20.1 years; 58.3% were African-American, 32.5% Caucasian. 9.3% other | Screening [Computerized Survey; Drug Abuse Treatment Outcome Studies, NIDA Quick Screen Question & Modified Alcohol, Smoking and Substance Involvement Screening Test (ASSIST)] | Six trajectory groups for non-partner violence were identified: Non-violent (39.7%), low baseline initiators (5.8%), moderate baseline persistent (21.0%), moderate baseline desistance (18.0%), high baseline desistance (9.5%), and high baseline persistent (5.8%). Binge drinking and cannabis, illicit drug, nonmedical prescription stimulant, and polysubstance use in the 30 days prior to their index ED visit were associated with increased likelihood of medium to high non-partner violence group membership in the two years following their index ED visit. |
| Jason E. Goldstick (2021) | Substance Use/Addiction | National Institute of Alcohol Abuse and Alcoholism, National Institute on Drug Abuse | 4 | To determine within-and between-person factors related to changes in drug use disorder diagnosis | Retrospective Cohort Study | Patients aged 14-24 years presenting to Hurley Medical Center ED in Flint, MI for assault-related injury and a non-assault-related injury group proportionally balanced by sex and age who self-reported any drug use | n=599 participants; 58.8% male; mean age 20.1 years; 58.3% were African-American, 32.5% Caucasian. 9.3% other | Screening [Computerized Survey; Drug Abuse Treatment Outcome Studies, NIDA Quick Screen Question & Modified Alcohol, Smoking and Substance Involvement Screening Test (ASSIST)] | 57.1% had a diagnosis of drug use disorder during their baseline ED visit; Positive (aOR 0.81 [95%CI 0.70-0.94]) and negative (aOR 1.73 [95%CI 1.45-2.07]) peer behaviors were associated with a drug use disorder diagnosis; community violence/crime (aOR 1.28 [95%CI 1.14-1.44]) and alcohol use quantity (aOR 1.06 [95%CI 1.02-1.11]) were associated with drug use disorder diagnosis, with primarily within-person effects; interpersonal violence involvement (aOR1.47 [95%CI 1.21-1.78]) showed both within- and between-person effects |
| Adam D. Laytin  (2017) | Emergency Medicine | Not Listed | 3 | To explore the prevalence of risk factors for violent injury among younger (age < 30 years) and older (age 30 $\geq$ years) victims of violent injury and to determine the long-term mortality rates in these age groups and explore the association between risk factors for violent injury and long-term mortality | Retrospective Cohort Study | Patients aged $\geq$ 18 years presenting to San Francisco General Hospital ED for medical treatment for violent injury recruited as a part of a larger randomized controlled trial to evaluate the impact of trauma-informed mental health services | n=541 participants; 75.0% male; median age 37 years; 52.0% African-American, 21.0% White, 12.0% Latino, 15.0% Other | Screening [Survey; Average weekly substance use] | 58% of participants reported illicit drug use; no difference in illicit drug use when comparing younger vs. older adults (62% vs 56%, p=0.21) |
| Patrick M. Carter (2020) | Public Health | National Institute on Drug Abuse and National Institute for Advancing Translational Sciences | 4 | To describe the rates/characteristics of risky firearm behaviors among urban adolescents and young adults and to examine risk/protective factors associated with risky firearm behaviors | Cross-Sectional Study | Patients aged 16-29 presenting to Hurley Medical Center ED in Flint, MI for any reason as a part of an intensive longitudinal study of firearm behaviors conducted among adolescents and young adults seeking urban ED treatment | n=1312 participants; 29.6% male; mean age 22.2 years; 50.5% were Black | Screening [Computerized Survey; Modified Alcohol, Smoking and Substance Involvement Screening Test (ASSIST)] | 102 (7.8%) reported engaging in risky firearm behaviors; of those engaging in risky firearm behaviors, 42 (41.2%) reported engaging in any firearm aggression; substance misuse (aOR 2.75 [95%CI 1.54-4.92]) was correlated with risky firearm behaviors |
